# Rectum to Medulla Oblongata: Colorectal Cancer Metastasizing to the Brainstem

**DOI:** 10.7759/cureus.39738

**Published:** 2023-05-30

**Authors:** Rachaita Lakra, Philip Bouchette, Milin Rana, Shreedhar Kulkarni

**Affiliations:** 1 Internal Medicine, Louisiana State University Health Sciences Center, Shreveport, USA; 2 Radiology, Louisiana State University Health Sciences Center, Shreveport, USA

**Keywords:** palliative and supportive care, carcinoembryonic antigen, central nervous system involvement, brainstem metastasis, colorectal cancer

## Abstract

Metastasis with colorectal cancer (CRC) is commonly seen in the liver, lungs and peritoneal cavity. Brainstem involvement with CRC is not studied with no prior reported cases. We report a case of CRC, admitted for apneic spells and dry cough and later found to have metastasis to the left anterolateral medulla oblongata. A 28-year-old male, with a past medical history of asthma, and colorectal adenocarcinoma metastatic to the brain, presented to the emergency department with complaints of a dry cough, altered mental status and shortness of breath. He was seen at urgent care before and was given a week of oral levofloxacin for presumptive pneumonia without any relief. Physical examination was concerning for stridor with clear lung fields. MRI brain showed previously noted post-operative right frontoparietal craniotomy changes and a new 9 x 8 x 8 mm ring-enhancing intra-axial lesion centered at the left anterolateral medulla oblongata indicative of brainstem metastatic disease. The patient was intubated for airway protection and underwent a suboccipital craniotomy for resection of the left pontomedullary mass, and histopathology was positive for metastatic adenocarcinoma, colorectal primary with hemorrhagic necrosis. He had a tracheostomy placed post multiple failed extubation trials and a gastrostomy tube for oral feeds. Goals of care were addressed with the patient and family, and a decision was made for home hospice.

## Introduction

Colorectal cancer (CRC) remains the third most common malignancy and the third leading cause of cancer mortality in the United States with an 8% prevalence in women and 9% in men [1]. Common metastatic sites include the liver (30%-40%), lungs (15%-20%) and peritoneal cavity (< 10%), with hepatic metastasis being the most common and the predominant cause of mortality [2]. The involvement of the central nervous system (CNS) is rare with CRC, therefore not elaborately studied. Previous literature reports an incidence of CNS metastasis in CRC as 1% to 4% [3]. With the advancement in therapeutic approach, the median survival of CRC has increased to 21-24 months, attributing to increased cases of CNS metastasis being reported [4].

Historically, management of CNS metastasis from CRC was mainly targeted at the palliative approach; however, with the advent of advanced therapies, the overall prognosis has improved. Most brain metastases from solid tumors use the hematogenous route predominantly involving the cerebrum (80%), followed by the cerebellum (15%), and rarely the brainstem (5%) [5]. Brainstem involvement with CRC, specifically rectal adenocarcinoma, is not studied with no prior reported cases other than autopsy studies. We report a case of rectal adenocarcinoma, admitted for apneic spells and dry cough and later found to have metastasis to the left anterolateral medulla oblongata in a young patient.

## Case presentation

A 28-year-old Caucasian male, with a past medical history of asthma and colorectal adenocarcinoma metastatic to the brain, presented to the emergency department with complaints of a dry cough, shortness of breath and altered mental status. He was seen at urgent care before and was given a week of oral antibiotics for presumptive pneumonia without any relief. Physical examination was concerning for stridor with otherwise clear lung fields.

The patient was diagnosed with metastatic rectal adenocarcinoma six months prior to index admission, after being admitted to the hospital for headaches, dizziness and blurred vision. He had no past family history of CRC. He was found to have a solitary mass-like lesion in the right inferior frontal gyrus on brain magnetic resonance imaging (MRI) and underwent craniotomy with resection of the mass. The histopathology resulted in metastatic CRC. Computed tomography (CT) was significant at a 2.7 x 3.9 x 7.1 cm mass in the right anterolateral wall of the rectum. The patient was initiated on chemotherapy with capecitabine and oxaliplatin. Panitumumab was later added due to Kirsten rat sarcoma viral oncogene homolog (KRAS) mutation and neuroblastoma RAS viral oncogene homolog (NRAS) wild type on the next gene sequencing. Laboratory workup resulted in hemoglobin of 9.7 g/dL, white blood cells of 7.78 K/µL, platelets of 119 K/µL, sodium of 123 mmol/L (other causes of hyponatremia were ruled out, and syndrome of inappropriate antidiuretic hormone ADH release (SIADH) was considered the cause), potassium of 3.9 mmol/L, bicarbonate of 22 mmol/L, alkaline phosphatase of 74 U/L, total bilirubin of 2.2 mg/dL, aspartate aminotransferase of 44 U/L, alanine transaminase of 28 U/L, carcinoembryonic antigen (CEA) of 13.7 ng/mL, alpha-fetoprotein of 1.9 ng/mL and lactic acid of 1.4 mmol/L (Table 1).

**Table 1:** Patient's laboratory workup at admission. CEA: carcinoembryonic antigen.

Laboratory Name	Patient’s Results	Reference Range
Hemoglobin	9.7 g/dL	12.5-16.3 g/dL
White blood cells	7.78 K/µL	3.9-12.70 K/µL
Platelets	119 K/µL	50-450 K/µL
Sodium	123 mmol/L	136-145 mmol/L
Potassium	3.9 mmol/L	3.5-5.1 mmol/L
Bicarbonate	22 mmol/L	23-29 mmol/L
Alkaline phosphatase	74 U/L	55-135 U/L
Total bilirubin	2.2 mg/dL	0.1-1 mg/dL
Aspartate aminotransferase	44 U/L	10-40 U/L
Alanine transaminase	28 U/L	10-44 U/L
CEA	13.7 ng/mL	0.0-5.0 ng/mL
Alpha-fetoprotein	1.9 ng/mL	0.0-8.4 ng/mL
Lactic acid	1.4 mmol/L	0.5-2.2 mmol/L

**Figure 1:**
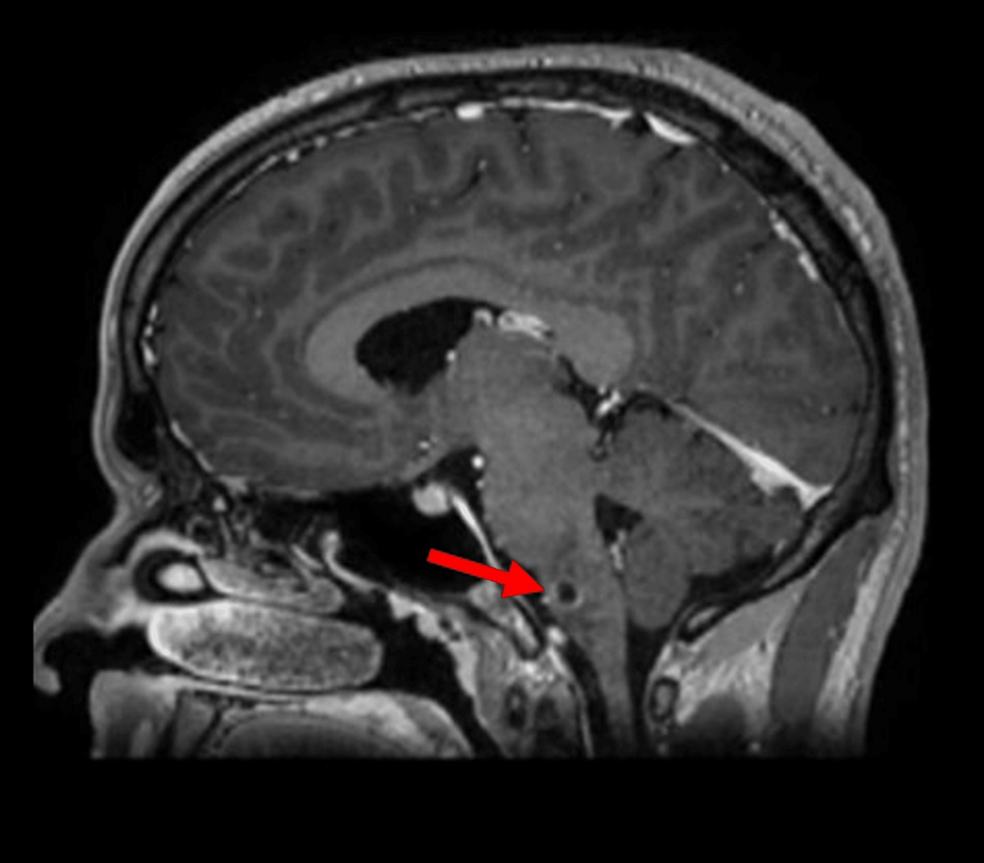
Sagittal T1-weighted contrast-enhanced brain MRI demonstrating a hypointense ring-enhancing lesion at the left medulla oblongata (arrow).​

**Figure 2:**
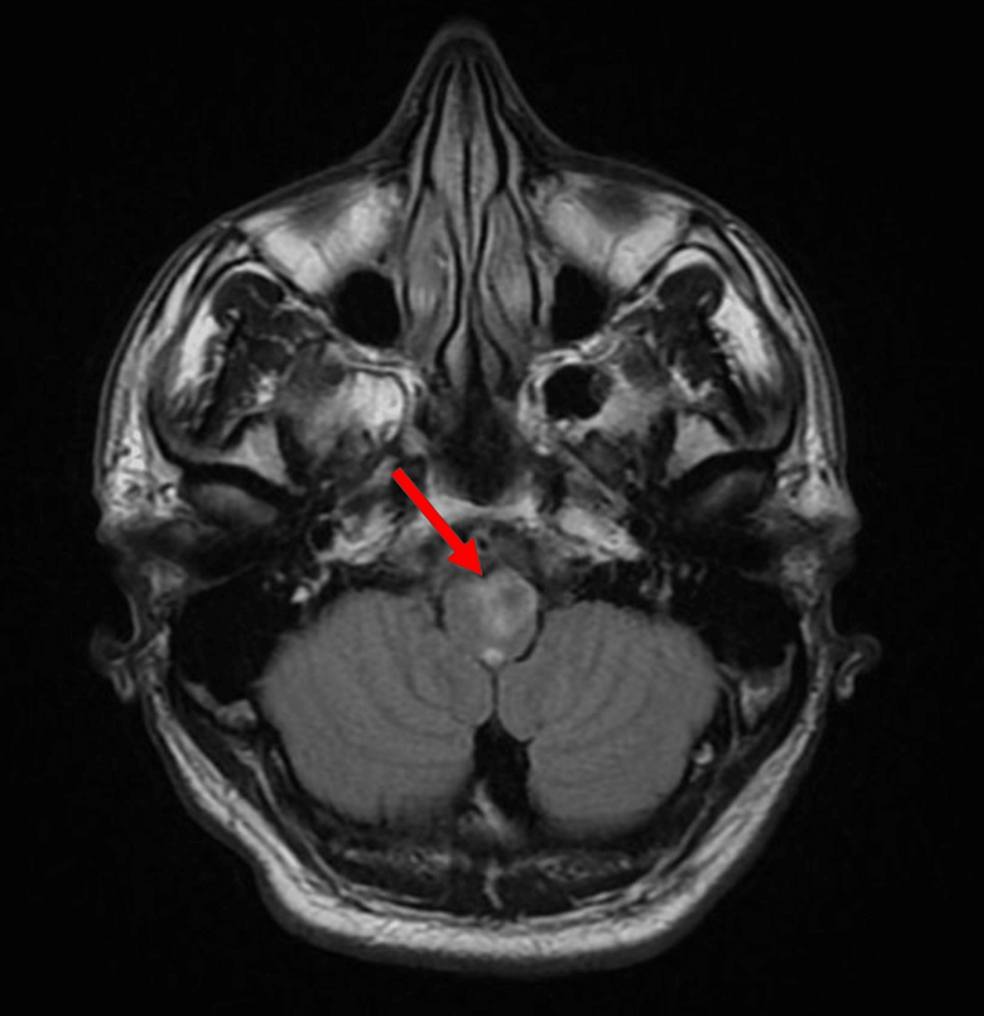
Axial T2 fluid-attenuated inversion recovery (FLAIR) demonstrating a new ring-enhancing lesion at the left medulla oblongata (arrow) associated with a halo of vasogenic edema​.

**Figure 3:**
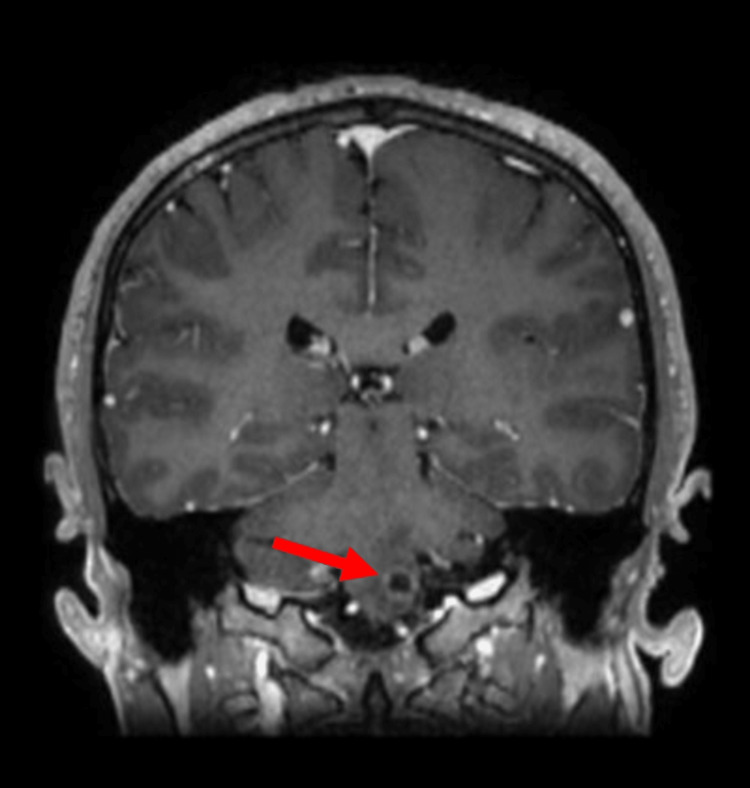
Coronal T1-weighted contrast-enhanced brain MRI demonstrating a hypointense ring-enhancing lesion at the left medulla oblongata (arrow).

Otolaryngology was consulted, and a barium swallow study was suggestive of impaired motility making the patient at high risk for aspiration. Over the next day, the patient had worsening encephalopathy and hypoxia, with high oxygen requirements on a 100% fraction of inspired oxygen (FiO_2_) using a high-flow nasal cannula with intermittent apneic spells. MRI of the brain showed previously noted post-operative right frontoparietal craniotomy changes and a new 9 x 8 x 8 mm ring-enhancing intra-axial lesion centered at the left anterolateral medulla oblongata, associated with vasogenic edema indicative of brainstem metastatic disease (Figures 1-3).

The palliative care team was consulted; however, the patient and family wanted all active treatment measures. The patient was intubated for airway protection and underwent a suboccipital craniotomy for resection of the left pontomedullary mass. Histopathology results were positive for metastatic rectal adenocarcinoma. He had a tracheostomy placed post multiple failed extubation trials and a percutaneous gastrostomy tube for oral feeds.

During the course of hospitalization, he reported abdominal pain, and the CT scan was significant for a large pneumoperitoneum, with multiple focal lesions in bilateral hepatic lobes and a moderate amount of heterogenous intraperitoneal fluid. He then was taken for an exploratory laparotomy, intraoperative findings included hepatic flexure perforation and multiple abscesses, and right hemicolectomy was performed. The patient’s hospital course was also complicated by pneumonia, with cultures from bronchoalveolar lavage growing *Staphylococcus epidermidis*, treated with vancomycin, clindamycin and additionally metronidazole and had a pleural effusion requiring chest tube placement. Goals of care were re-addressed with the patient and family, and a shared decision was made to focus on keeping him more comfortable with no further invasive procedures and was discharged to home hospice.

## Discussion

Brain metastases by solid tumors outnumber primary brain tumors [6]. The overall reported incidence of CNS metastasis from CRC has been low in both clinical and autopsy literature [7]; however, previous studies have postulated that longer survival with CRC is due to the advances in therapies with cytostatic drugs and biological agents [8]. Per data from past literature, the distal colon including the rectum (48%) and sigmoid colon (12%) is relatively more frequently seen as a site for brain metastasis than the proximal colon (40%) similar to our patient’s case of rectal cancer [9].

The pathophysiology of CRC metastasis is poorly understood, with some authors supporting the “homing” mechanism [10]. Delattre et al. narrated communication between pelvic and vertebral veins (Batson's vertebral plexus) as a potential route for metastatic emboli, during periods of increased intra-abdominal pressure [11]. Cytogenetics for CRC metastasis has not been consolidated yet since reticular activating system (RAS) mutations are known to be associated with brain metastases; however, it does not explain the higher association of rectal CRC with brain metastasis, since RAS mutations are more frequently seen in right colon CRC versus rectum [12]. The CXCR4/CXCL12 axis, a chemokine/receptor pair, is expressed by cancer cells in CRC, highly expressed with rectal tumors [13]. CXCR4 expression is previously correlated with distant metastasis and overall survival [14]. Mongan et al. reported an association between CXCR4/CXCL12 and brain metastasis in CRC, which could be of prognostic significance [15].

CRC with brain metastasis is associated with increased morbidity and poor survival rates. The outlined median survival ranges between 2.6 and 8 months, with very limited cases surviving more than a year [16]. Alden et al. reported a median survival of less than three months in patients with CNS metastasis from CRC [17], similar to four months described by Damiens et al. [18]. There remains limited research on risk factors to develop CNS involvement in CRC. Christensen et al. reported male sex, rectal CRC and previously reported lung metastasis as important indicators for the likelihood of brain metastasis [19]. CEA, an established tool for monitoring therapy, is seen in approximately 70% of patients with CRC and has a limited metastatic predictive role [20]. Our patient was a young male with rectal adenocarcinoma and had high CEA levels. Increased surveillance of risk factors and a low threshold for brain imaging can help with early detection and may improve overall survival. The unique aspect of this case is the concurrent diagnosis of rectal carcinoma with brain metastasis, specifically medulla oblongata, in a young patient with no family history of CRC, which to our best knowledge has not been reported in the literature. Another challenging aspect of this case was the age of our patient (28 years), requiring difficult goals of care conversations and finally leading to hospice care.

## Conclusions

Although there has been significant improvement in the median survival of colorectal carcinoma in recent years with advanced therapeutic approaches, the disease burden of brain metastasis is further increased with high mortality and morbidity; therefore, understanding the risk factors and prognostic outcomes is crucial. Our patient had a cough and apneic episodes, and later imaging proved medulla involvement, indicating that a low threshold for brain imaging should be considered for any signs and symptoms of central nervous system metastasis. This case report contributes to the limited literature on brain metastasis from colorectal cancer, specifically to the medulla oblongata in a young patient. 
